# Taurine transport in human placental trophoblast is important for regulation of cell differentiation and survival

**DOI:** 10.1038/cddis.2013.81

**Published:** 2013-03-21

**Authors:** M Desforges, L Parsons, M Westwood, C P Sibley, S L Greenwood

**Affiliations:** 1 Maternal and Foetal Health Research Centre, Institute of Human Development, University of Manchester, St. Mary's Hospital, Central Manchester University Hospitals NHS Foundation Trust, Manchester Academic Health Science Centre, Level 5-Research, Manchester, UK

**Keywords:** TauT, SLC6A6, system beta, differentiation, apoptosis, syncytiotrophoblast

## Abstract

The outer epithelial cell layer of human placenta, the syncytiotrophoblast, is a specialised terminally differentiated multinucleate tissue. It is generated and renewed from underlying cytotrophoblast cells that undergo proliferation, differentiation and fusion with syncytiotrophoblast. Acquisition of fresh cellular components is thought to be balanced by apoptosis and shedding of aged nuclei. This process of trophoblast cell turnover maintains a functional syncytiotrophoblast, capable of sufficient nutrient transfer from mother to foetus. Foetal growth restriction (FGR) is a pregnancy complication associated with aberrant trophoblast turnover and reduced activity of certain amino acid transporters, including the taurine transporter (TauT). Taurine is the most abundant amino acid in human placenta implying an important physiological role within this tissue. Unlike other amino acids, taurine is not incorporated into proteins and in non-placental cell types represents an important osmolyte involved in cell volume regulation, and is also cytoprotective. Here, we investigated the role of taurine in trophoblast turnover using RNA interference to deplete primary human trophoblast cells of TauT and reduce intracellular taurine content. Trophoblast differentiation was compromised in TauT-deficient cells, and susceptibility of these cells to an inflammatory cytokine that is elevated in FGR was increased, evidenced by elevated levels of apoptosis. These data suggest an important role for taurine in trophoblast turnover and cytoprotection.

The outermost cell layer of the human placenta, the syncytiotrophoblast, is a specialised multinucleate tissue that functions as a solute-transporting epithelium and endocrine/paracrine organ, delivering nutrients to the foetus and producing hormones that sustain pregnancy. Consequently, maintenance of the syncytiotrophoblast is vital for a successful pregnancy. The syncytiotrophoblast exists in a terminally differentiated post-mitotic state; it is generated and renewed from the underlying population of mononuclear cytotrophoblast cells, which undergo proliferation, differentiation and finally fusion with the syncytiotrophoblast, permitting the acquisition of fresh cellular components. It is hypothesised that apoptosis and subsequent shedding of synctial knots into the maternal circulation complete the process of syncytiotrophoblast nuclear turnover.^1^ However, there is an alternative hypothesis proposed whereby syncytial nuclei continue to accumulate until the end of pregnancy.^2^

Despite the controversy surrounding the life cycle of apoptotic nuclei during normal trophoblast turnover, it is agreed that there is increased trophoblast cell death in placentas from pregnancies complicated by foetal growth restriction (FGR).^3^ Low birth weight is associated with an increased risk of neonatal morbidity and mortality, and development of metabolic and cardiovascular diseases in adulthood.^4^ Ongoing research investigating the cause(s) of altered trophoblast turnover in FGR has identified a number of factors that could trigger inappropriate apoptosis, including elevated inflammatory cytokines such as tumour necrosis factor alpha (TNF*α*).^5^

Abnormal turnover compromises syncytiotrophoblast integrity and renewal with consequences for nutrient delivery to the foetus, and this is thought to be a major contributing factor to FGR. Consistent with this is the finding that in addition to aberrant trophoblast turnover in FGR, there is reduced activity of certain amino acid transporters in the syncytiotrophoblast including that of system *β*^6^ responsible for taurine uptake into the placenta.

Taurine is a non-essential amino acid, as it can be synthesised from methionine and serine that form its precursor, cysteine. However, during foetal life, taurine is essential because the human foetus and placenta lack the necessary synthetic enzymes.^7^ Therefore, foetal and placental demand for taurine must be met by transport of taurine from maternal plasma into the syncytiotrophoblast and across the placenta via system *β*.^8^

Foetal plasma taurine concentrations are lower in FGR compared with normal pregnancies,^9^ suggesting taurine to be important for foetal growth. Unlike other amino acids, taurine is not incorporated into proteins and is largely involved in promoting development of the central nervous system, retina, kidney, and endocrine pancreas.^10, 11^

Taurine is the most abundant free amino acid in human placenta (intracellular concentration ∼10 mM, maternal and foetal plasma 60 and 135 *μ*M, respectively).^12^ The reason why intracellular taurine levels are so high in the syncytiotrophoblast is unknown, but could reflect the cytoprotective functions of taurine and also its role as an osmolyte, important for cell volume regulation, as found in other cell types.^13^ All cells have to regulate their volume in order to survive; macromolecular synthesis, cell growth, differentiation, apoptosis and hormone secretion are all influenced by the cellular hydration state.^14^

We therefore hypothesised that as well as being directly important for foetal development, system *β*-mediated taurine transport by the placenta indirectly affects foetal growth by maintaining the normal process of trophoblast cell turnover. We further hypothesised that reduced taurine uptake by syncytiotrophoblast could compromise cytoprotection against TNF*α*, an inflammatory cytokine that is elevated in FGR, and increase susceptibility to inappropriate apoptosis.

These hypothesese have been tested by investigating cytotrophoblast differentiation and survival following exposure to TNF*α in vitro* after siRNA-mediated knockdown of the system *β* amino acid transporter protein, taurine transporter (TauT) (encoded by the *SLC6A6* gene). Primary cytotrophoblast cells isolated from term human placenta were used as a model for investigating trophoblast cell turnover because, when maintained in culture, these mononucleate cells are mitotically inactive, and therefore unable to proliferate. Instead, the cytotrophoblast cells aggregate at 18–24 h of culture and subsequently fuse to form multinucleated cells, reminiscent of the process of differentiation into syncytiotrophoblast *in vivo*.^15^

## Results

### siRNA-mediated knockdown of *SLC6A6*/TauT

**Figure 1:**
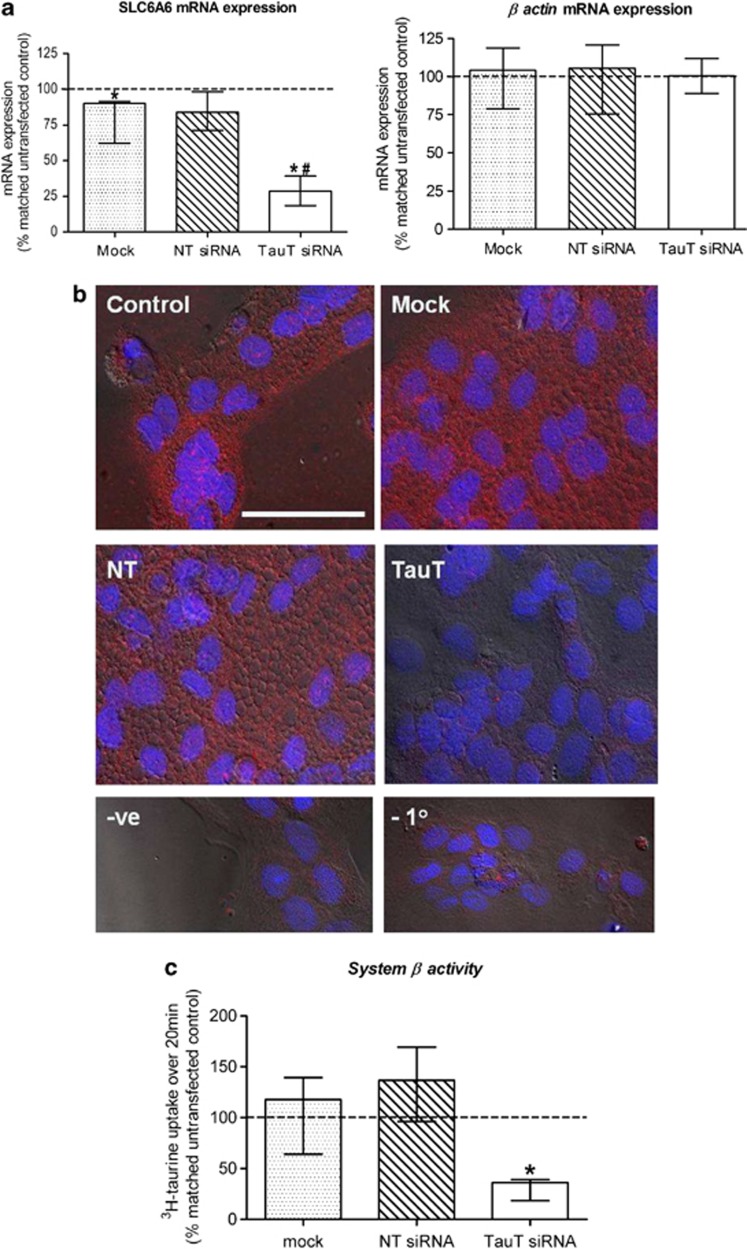
Confirmation of TauT knockdown in 66-h cytotrophoblast cells. (**a**) *SLC6A6* and *β actin* mRNA expression. (**b**) Immunofluorescent detection of TauT protein (red) in cells counterstained with DAPI (blue). Scale bar represents 50 *μ*M and refers to all images. A lack of fluorescence following pre-absorption of primary antibody with a 10 × excess of antigenic peptide (−ve) or omission of the primary antibody (−1°) confirmed specificity. (**c**) TauT activity. All observations were made 48 h post transfection. Key to labelling: control=untransfected, mock=transfection reagent only, NT=non-targeting siRNA, TauT=*SLC6A6*-specfic siRNA. Error bars represent median±interquartile range, *n*=5. *P<0.05 *versus* 100% (i.e., matched untransfected control), Wilcoxon-signed Rank test. ^#^*P*<0.05 *versus* mock and NT siRNA, Kruskal–Wallis with Dunn's multiple comparison test

Transfection of cytotrophoblast cells with 50 nM *SLC6A6*-specific siRNA significantly reduced target mRNA expression by 71% (median, range: 64–84%, *n*=5) when compared with untransfected control cells (Figure 1a). Expression of mRNA for the housekeeping gene, *β actin*, was unaffected by transfection with *SLC6A6*-specific siRNA (Figure 1a), indicating that this siRNA does not cause off-target/non-specific effects. Transfection of cytotrophoblast cells with 50 nM non-targeting siRNA did not affect mRNA expression for *SLC6A6* nor *β* actin (Figure 1a). Exposure of cytotrophoblast cells to DharmaFECT 2 transfection reagent alone (mock transfected) caused a small but significant decrease in *SLC6A6* mRNA expression when compared with untransfected controls cells (Figure 1a). Despite reduced expression of *SLC6A6* mRNA in mock transfected cells, TauT protein expression and activity in these cells was comparable to untransfected controls (Figures 1b and c). *SLC6A6* mRNA knockdown in cells transfected with 50 nM *SLC6A6*-specific siRNA was accompanied by reduced TauT protein expression, evidenced by diminished immunofluorescent detection of this target protein when compared with untransfected and mock transfected control cells, as well as cells transfected with 50 nM non-targeting siRNA (Figure 1b). System *β* amino acid transporter/TauT initial rate activity was significantly reduced by 64% (median, range: 60–82%, *n*=5), following *SLC6A6*/TauT knockdown (Figure 1c). Successful knockdown of TauT expression and activity in the primary cytotrophoblast cells was therefore achieved.

### TauT knockdown reduces intracellular taurine accumulation by cytotrophoblast cells

**Figure 2:**
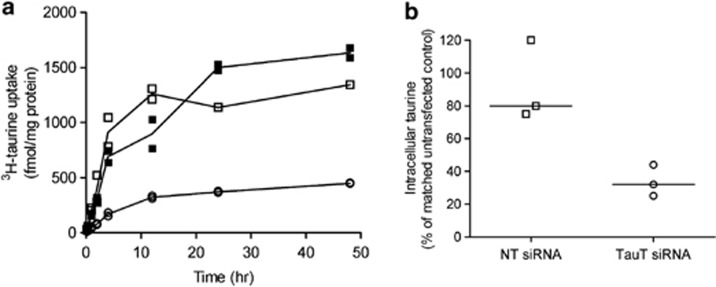
TauT knockdown reduces intracellular taurine accumulation. (**a**) Cellular accumulation of taurine (tracer concentration of ^3^H-taurine +9 *μ*M unlabelled taurine) reaches steady state after 24 h in control cells (closed squares) and cells treated with non-targeting (open squares) or *SLC6A6*-specific (open circles) siRNA. Duplicate measurements from one placenta. (**b**) Scatter plot of intracellular taurine accumulation after 24 h. Each point represents the mean of duplicate observations of three placentas (line represents median). NT=non-targeting siRNA, TauT=*SLC6A6*-specfic siRNA

Intracellular taurine accumulation by untransfected control cytotrophoblast cells reached steady state after 24 h (Figure 2a). As taurine is not metabolised, this accumulation reflects the stable intracellular taurine concentration when uptake via TauT and efflux out of the cell (thought to occur via volume-regulated anion channels^16^) are at steady state. Similarly, in cells transfected with 50 nM non-targeting or *SLC6A6*-specific siRNA, steady state occurred after 24 h (Figure 2a). However, the ability of the cytotrophoblast cells transfected with *SLC6A6*-specific siRNA, and therefore with reduced TauT expression and activity, to accumulate intracellular taurine was severely compromised (∼33% of that achieved by control cells at 24 h, Figure 2b). Taurine uptake was allowed to occur over a total period of 48 h but, even by this time point, the TauT-deficient cells were unable to accumulate any additional intracellular taurine (Figure 2a). Total protein content of cells transfected with *SLC6A6*-specific siRNA did not decline during this prolonged period of culture and was comparable to total cell protein content of untransfected controls and cells transfected with non-targeting siRNA at the 48 h time point (data not shown). This indicates that cell number was unaffected by transfection, even over a prolonged period, and a fall in the number of cells cannot underlie the reduced ^3^H-taurine accumulation. These observations demonstrate that reduced TauT activity in cytotrophoblast cells (Figure 1c: initial rate) is associated with lower intracellular taurine levels at steady state.

### Trophoblast differentiation is impaired following TauT knockdown

**Figure 3:**
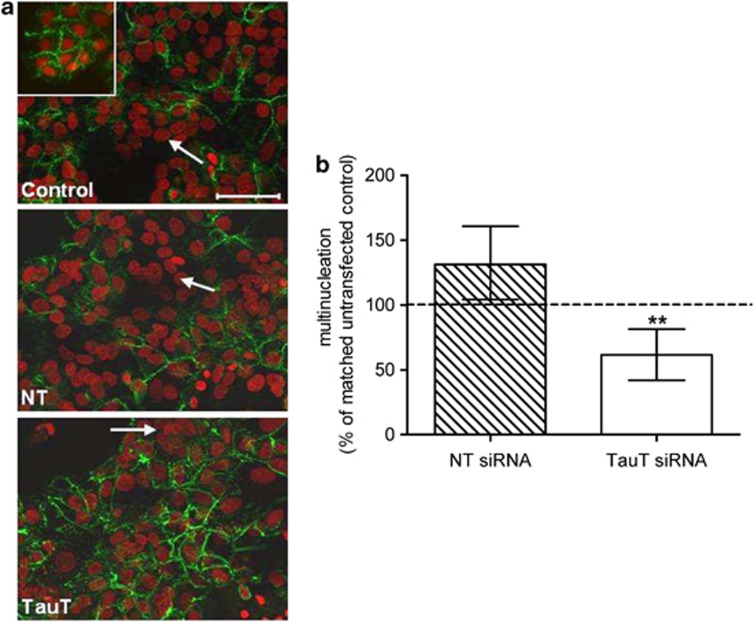
TauT knockdown impairs cytotrophoblast morphological differentiation *in vitro*. (**a**) Immunofluorescent detection of desmosomes (green) in 18 h (inset) and 66 h cytotrophoblast cells counterstained with propidium iodide (red). The white arrows indicate multinucleated cells (defined as ≥3 nuclei within desmosomal boundaries). (**b**) Percentage of multinucleated cells at 66 h following transfection, relative to matched control cells (*n*=7, error bars represent median±interquartile range). **P<0.01 *versus* 100% Wilcoxon-signed Rank test. NT=non-targeting siRNA, TauT=*SLC6A6*-specfic siRNA

Desmosomes are specialised epithelial cell–cell junctions between adjoining cells.^17^ As cytotrophoblast cells differentiate and become multinucleated, the loss of cell–cell boundaries parallels a decrease in desmosomal proteins. Untransfected cytotrophoblast cells differentiated normally during the 66 h in culture, evidenced by reduced immunofluorescent staining of desmosomal proteins (Figure 3a, 18 *versus* 66 h control). This morphological differentiation was accompanied by a significant increase in human chorionic gonadotrophin (hCG) secretion over time in culture from 2.2 mIU/mg protein/h at 18 h to 29 and 318 mIU/mg protein/h at 42 and 66 h, respectively (mean, *n*=6. *P*<0.001 Kruskal–Wallis test), consistent with concomitant biochemical differentiation.

There was significantly less multinucleation at 66 h in cells transfected with *SLC6A6*-specific siRNA, suggesting a role for TauT-mediated taurine transport and/or intracellular taurine in trophoblast morphological differentiation (Figures 3a and b). Transfection of cells with non-targeting siRNA did not affect multinucleation (Figures 3a and b), eliminating the possibility that impaired trophoblast morphological differentiation was an effect of the transfection procedure itself. However, reduced morphological differentiation of cytotrophoblast cells following TauT knockdown was not associated with reduced biochemical differentiation, as there was no significant difference in hCG secretion by untransfected control cells and cells transfected with *SLC6A6*-specific siRNA at 66 h (*n*=6, data not shown).

### Effects of TauT knockdown on apoptosis

**Figure 4:**
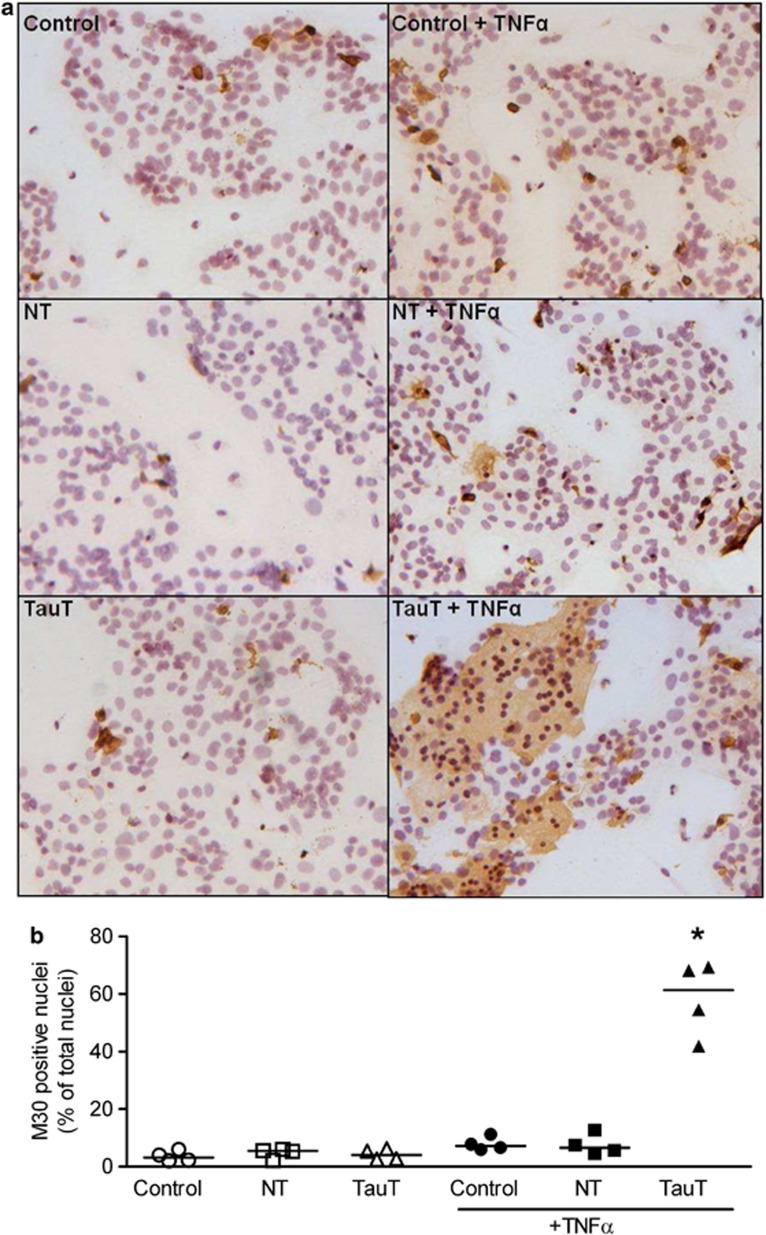
TauT-deficient cytotrophoblast cells are more susceptible to TNF*α*-induced apoptosis. (**a**) Detection of apoptosis in primary cytotrophoblast cells using positive staining for caspase-cleaved cytokeratin 18 (M30 CytoDEATH). Counterstained with hematoxylin. M30 staining (brown) appears in the cytoplasm of apoptotic cells. (**b**) Scatter plot of M30-positive cells expressed as a percentage of total number of nuclei (*n*=4, line represents the median). *P<0.05 Kruskal–Wallis with Dunn's post test. NT=non-targeting siRNA, TauT=*SLC6A6*-specfic siRNA, +TNF*α*=overnight treatment with 100 ng TNF*α*

Here, we investigated if TauT/intracellular taurine has a role in regulating cell survival by assessing basal and TNF*α*-induced apoptosis in cytotrophoblast cells following TauT knockdown. Apoptotic cell death was determined by immunohistochemical detection of a caspase-cleaved fragment of cytokeratin 18 (M30) that is produced during apoptosis. In untransfected cytotrophoblast cells, 2–6% of syncytialised cells were apoptotic at 66 h of culture (Figure 4b). Transfection with non-targeting or *SLC6A6*-specific siRNA had no significant effect on this basal level of apoptosis (Figure 4b). However, overnight treatment of cytotrophoblast cells with 100 ng of the proinflammatory cytokine TNF*α* significantly increased apoptosis but only in those cells with siRNA-mediated TauT knockdown (Figures 4a and b).

## Discussion

Our observations provide the first evidence that TauT-mediated taurine transport in human placenta is important for the normal process of trophoblast turnover. More specifically, the ability of trophoblast cells to accumulate intracellular taurine facilitates their differentiation into a multinucleated syncytium and protects against inappropriate cell death in response to an inflammatory cytokine.

It is well documented that in both placental and non-placental cell types, taurine is a key osmoregulator with cytoprotective functions.^13, 18^ In order to carry out these important roles, it is essential that intracellular taurine concentration is maintained by appropriate regulation of cellular taurine uptake and efflux. In placentas from normal pregnancy, taurine is accumulated in the syncytiotrophoblast to reach levels of ∼10 mM.^12^ *In vitro* studies have demonstrated that TauT activity in the foetal-facing basal membrane of the syncytiotrophoblast is only 6% of that measured in the maternal-facing microvillous membrane (MVM),^6^ suggesting that the high concentration of taurine found within the syncytiotrophoblast is achieved by the uptake from maternal blood. Norberg *et al.*^6^ found that TauT activity in MVM of placentas from pregnancies complicated by FGR was reduced by 34% compared with normal pregnancies. In the current study, a 64% reduction in trophoblast TauT activity led to a 66% decrease in intracellular taurine accumulation. It is therefore probable that intracellular taurine levels are lower in the syncytiotrophoblast of placentas from FGR pregnancies, although this remains to be determined.

Our data demonstrate that TauT-mediated taurine transport in trophoblast cells has a role in their fusion/differentiation and multinucleation, but does not influence hCG secretion. This observation suggests that intracellular taurine has selective effects on these two well-characterized events in trophoblast differentiation, with maintenance of normal intracellular taurine being necessary for syncytialisation, but not for hormone secretion. This is consistent with previous reports that biochemical and morphological differentiation of trophoblast cells *in vitro* can be independent events.^17, 19^ Reduced placental TauT activity in FGR could, therefore, lead to impaired syncytial formation and renewal. Indeed, there is evidence that cytotrophoblast cells isolated from pregnancies with placental insufficiency and FGR have a significantly lower cell–cell fusion index compared with those isolated from normal placentas.^20^ Trophoblast fusion events and their regulation are poorly understood. Exactly how TauT-mediated taurine transport influences trophoblast cell fusion/multinucleation requires investigation but two mechanisms are proposed. Intracellular taurine could be important for intracellular signalling events, which facilitate morphological differentiation. In non-placental cells, intracellular taurine modulates the expression and phosphorylation of proteins involved in the MAPK, STAT3 and PKC signalling pathways.^21^ Each of these signalling molecules are involved in cell differentiation,^22, 23, 24^ therefore, alterations in intracellular taurine could affect their role in this process. Alternatively, taurine could help maintain gap junctional intercellular communication required for cell fusion, as has been shown in liver.^25^ Indeed, molecular exchanges through gap junctions preceding cellular fusion are essential for trophoblast differentiation and generation of the multinucleated syncytiotrophoblast.^26^

In addition to an impaired ability to differentiate morphologically, we have demonstrated that TauT-deficient trophoblast cells are more susceptible to TNF*α*-induced apoptosis. Elevated levels of TNF*α* have been reported in FGR and this has been suggested to contribute to the increase in trophoblast apoptosis associated with these pregnancy complications.^5, 27^ However, we have demonstrated that TNF*α* induces significant apoptosis in the cytotrophoblast cells only when their ability to accumulate intracellular taurine has been reduced through *SLC6A6*-specific knockdown. This observation is in agreement with studies in other cell types that have revealed cytoprotective functions of taurine. Interestingly, an increased ability to accumulate taurine via overexpression of *SLC6A6* attenuated cisplatin-induced apoptosis in a renal cell line,^28^ supporting the hypothesis proposed by Han *et al.*^29^ that *SLC6A6* acts as an antiapoptotic gene by facilitating TauT-mediated taurine accumulation. It is therefore proposed that it is the reduced placental TauT activity, together with elevated levels of TNF*α*, that contribute to increased trophoblast cell death in cases of FGR.

The data presented, which highlight the importance of TauT-mediated taurine transport for trophoblast fusion/differentiation and survival, provide good reason for identifying the cause(s) of reduced placental TauT activity in FGR. Placental expression of TauT is comparable between normal pregnancies and those complicated with FGR.^30^ Therefore, one possible explanation for reduced placental TauT activity in this pregnancy condition is post-translational modification of the TauT protein, causing a conformational change that reduces the affinity for ligands. Molecular cloning and characterisation of TauT has revealed the presence of several phosphorylation sites.^31^ Phosphorylation of TauT by protein kinase C decreases its affinity for taurine,^32^ and activation of this kinase in placental villous fragments and trophoblast cells leads to reduced TauT activity.^30, 33^ TauT can also be nitrated,^34^ a modification of tyrosine residues by reactive nitrogen species (RNS), and this could also underlie the decreased syncytiotrophoblast TauT activity in FGR. Tyrosine residues are essential for the activity of TauT in human placenta^35^ and *in vitro* experiments suggest that nitrative stress impairs placental TauT activity.^30, 36^ Levels of RNS are elevated in FGR^37^ and TauT nitration could be increased as a consequence, but this remains to be investigated. There is evidence that an increased proportion of TauT is nitrated in placentas from pregnancies complicated with pre-eclampsia (V Roberts and L Myatt, personal communication). Pre-eclampsia is a serious disease of pregnancy sometimes accompanied by FGR that is also associated with abnormal trophoblast turnover:^3^ It will be interesting to determine whether syncytiotrophoblast TauT activity is also reduced in this pregnancy complication.

In summary, we have demonstrated that reduced TauT activity in cytotrophoblast cells leads to impaired morphological differentiation and an increased susceptibility to apoptotic cell death. Such alterations *in utero* would have implications for syncytiotrophoblast renewal, compromising the transfer of nutrients from the maternal circulation towards the foetus via other transporters present on the MVM ,and also cause disruption to the endocrine function of the syncytiotrophoblast, leading to placental insufficiency with consequences for foetal growth and wellbeing. The ability of trophoblast cells to accumulate sufficient intracellular taurine for their differentiation and survival is therefore crucial for a healthy pregnancy.

## Materials and Methods

### Materials

Unless stated otherwise, all materials used were obtained from Sigma-Aldrich (Poole, UK).

### Primary cytotrophoblast cell isolation and culture

Term placentas (38–40 weeks gestation) were collected with written informed consent and in accordance with the Local Ethics Committee's approval, following caesarean section or vaginal delivery from uncomplicated singleton pregnancies. Cytotrophoblast cells were isolated using an adaptation of the method used by Kliman *et al.*,^15^ as previously described.^38^ Cells destined for amino acid transporter activity measurements were plated onto 35-mm culture dishes (Nunc) at a density of 2–2.5 × 10^6^. Cells destined for mRNA analysis and detection of target proteins were plated into 12-well culture plates (Nunc) at a density of 1–1.5 × 10^6^. Cytotrophoblast cells were maintained for 66 h in culture medium (Dulbecco's modified Eagle's medium (DMEM) and Ham's F-12 1 : 1, 10% FCS (heat-inactivated), 1% gentamicin, 0.6% glutamine, 0.2% penicillin, 0.2% streptomycin) at 37 °C in a humidified incubator (95% air/5% CO2) supplemented with 100 *μ*M taurine to mimic physiological levels in maternal blood.^12^

### Transfection of primary cytotrophoblast cells with siRNA

Previous studies have demonstrated that at 18 h of culture, isolated cytotrophoblast cells are mononucleate and by 42 h they aggregate and fuse. At 66 h, the cells have differentiated to form large polarised multinucleate cells that resemble syncytiotrophoblast *in vivo*.^15, 38^ At 18 h of culture, cytotrophoblast cells were transfected with 50 nM *SLC6A6*-specific siRNA (Qiagen, West Sussex, UK) using DharmaFECT2-transfection reagent (Dharmacon, Fisher Scientific UK, Loughborough, UK), as described previously.^39, 40^ Cytotrophoblast cells transfected with non-targeting siRNA (Invitrogen, Life Technologies, Paisley, UK) and cells exposed to DharmaFECT2 only (i.e., mock transfected) were included as controls. Initially, four different *SLC6A6*-specific siRNAs were tested and here we present data using the construct, which most efficiently silenced *SLC6A6* (target sequence: 5′-CTGCTGTTTACTAACATTAGA-3′). In validation experiments, this construct reduced SLC6A6 mRNA expression by 64% compared with untransfected controls, whereas mRNA silencing by the other three constructs tested were 60, 50 and 27% (mean, *n*=2).

### Confirmation of target-specific mRNA knockdown using QPCR

48 h post transfection (i.e., at 66 h of culture), cells were lysed and total RNA extracted using an Absolutely RNA microprep kit (Stratagene, Agilent Technologies UK Ltd, Stockport, UK). RNA was quantified using a Quant-iT Ribogreen kit (Molecular Probes, Life Technologies), and 100 ng of total RNA from each sample was reverse transcribed using AffinityScript cDNA synthesis kit with random primers (Stratagene). mRNA for *β* actin and *SLC6A6* were quantified in a 1 : 10 dilution of the cDNA samples by QPCR using a Stratagene MX3000P real time PCR machine and Stratagene Brilliant SYBR Green I QPCR mastermix, with 5-carboxy-x-rhodamine as a passive reference dye. Primers (MWG-Biotech, Ebersberg, Germany) for *SLC6A6* were forward: 5′-CGTACCCCTGACCTACAACAAA-3′ and reverse: 5′-CAGAGGCGGATGACGATGAC-3′ (300 nM) designed using Beacon Designer software (Premier Biosoft Int., Palo Alto, CA, USA) and confirmed to be specific by BLAST assessment. Primers for *β* actin (200 nM) were as previously described.^40^ SLC6A6 and *β* actin mRNA were quantified against standard curves generated from human reference total RNA (Stratagene). Data were analysed by Wilcoxon-signed Rank test following normalisation of mRNA expression in the transfected cells to expression in the corresponding untransfected control cells for each experiment.

### Immunofluorescent staining

Cytotrophoblast cells that had been plated onto 16-mm glass coverslips in 12-well culture plates were fixed in methanol at −20 °C for 25 min and then stored at 4 °C in PBS prior to immunofluorescent staining for either TauT or desmosomal proteins (allowing visualisation of multinucleation^17^). Following a 30-min incubation at 37 °C with blocking solution (2% FCS, 2% BSA and 0.1% Tween20 in PBS) to reduce nonspecific binding, cells were incubated for 1 h at room temperature with primary antibody (1 : 100 dilution in blocking solution). Primary antibodies were rabbit polyclonal anti-Taurine Transporter (Alpha Diagnostics, Source BioScience LifeSciences, Nottingham, UK) and monoclonal mouse anti-desmosomal protein (D1286, mouse IgG1 isotope). Cells were then washed with PBS and the secondary antibody, AlexaFluor-568 goat anti-rabbit IgG_1_ (1 : 600 in block solution; Molecular Probes) and goat anti-mouse IgG FITC (F2012, 1 : 100 dilution), respectively, applied for 1 h at 37 °C in the dark. After washing with PBS, coverslips were mounted onto glass microscope slides using Vectashield mounting medium containing 4',6-diamidino-2-phenylindole (DAPI) or propidium iodide (PI) nuclear counterstain (Vector Labs, Peterborough, UK). Immunofluorescent images were captured using a Zeiss AxioObserver Inverted Microscope.

### Amino acid transporter activity measurements and ability to accumulate intracellular taurine

48 h post transfection (i.e., at 66 h of culture), cytotrophoblast cells were washed free of cell culture medium using Tyrode's buffer (135 mM NaCl, 5 mM KCl, 1.8 mM CaCl_2_, 1 mM MgCl_2_, 10 mM HEPES, 5.6 mM glucose, pH 7.4). Na^+^-dependent uptake of radiolabelled taurine (^3^H-taurine, 1 *μ*Ci/ml; 50 nM) by control and transfected cytotrophoblast cells was then measured in duplicate as follows: uptake of ^3^H-taurine was carried out at 37 °C in either control or Na^+^-free Tyrode's buffer (135 mM choline chloride replaced NaCl, pH 7.4). Uptake was terminated after 20 min, determined in pilot experiments to represent initial rate, by washing cells in 25-ml ice-cold Tyrode's buffer over 1 min. Cells were then lysed in 1 ml 0.3 M NaOH and the lysate counted for *β* radiation. Cell lysate protein content (mg) was determined using a commercial kit (Bio-Rad Laboratories Ltd., Hemel Hampstead, UK). Uptake of radiolabelled taurine is expressed as pmol per mg protein over 20 min. The Na^+^-dependent component of ^3^H-taurine uptake, representing TauT-specific uptake, was calculated by subtracting ^3^H-taurine uptake in the absence of Na^+^ from uptake in the presence of Na^+^. Data were analysed by Wilcoxon-signed Rank test, following normalisation to Na^+^-dependent ^3^H-taurine uptake by the corresponding untransfected control sample for each experiment.

The long-term accumulation of taurine was also determined to estimate the intracellular taurine at steady state in control and transfected cytotrophoblast cells. ^3^H-taurine (1 *μ*C/ml) was added to the culture medium and the cells incubated for 0.5–48 h (5% CO_2_/ air; 37 °C). The cells were then washed, lysed in 0.3 M NaOH, counted for *β* radioactivity and analysed for protein content, as described above. Intracellular taurine was calculated per mg cellular protein using the specific activity of the isotope and taking into account the concentration of labelled (50 nM) and unlabelled (9 *μ*M) taurine in the culture medium.

### Assessment of cytotrophoblast cell morphological differentiation

Immunofluorescent images of cytotrophoblast cells stained for desmosomes and nuclei (see above) were used to assess multinucleation as a measure of morphological differentiation. Using a previously published method,^19^ three observers, blinded to the identity of the images, counted the total number of nuclei per field of view and the number of multinucleated cells (defined as ≥3 nuclei within desmosomal boundaries) by using Image Pro Plus software (MediaCybernetics, Buckinghamshire, UK). The number of multinucleated cells was then expressed as a percentage of the total number of nuclei within a given field of view. The average number of nuclei per field of view was 114 (range 57–354). For each experiment, three fields of view were analysed per treatment and each treatment was performed in duplicate. The mean of these observations was then calculated to provide a value of multinucleation in transfected and untransfected control cells for each experiment. Multinucleation of transfected cells was expressed as a per cent of mutinucleation of matched control cells for the corresponding experiment and analysed by Wilcoxon-signed Rank test.

### Assessment of cytotrophoblast cell biochemical differentiation

The *β* subunit of hCG is produced by terminally differentiated syncytiotrophoblast and is used as an indicator of cytotrophoblast differentiation in culture, following their isolation from term placenta.^19^ Culture medium was collected at 18, 42 and 66 h of cytotrophoblast cell culture, and stored in aliquots at −20 °C. The medium was assayed for secreted *β*-hCG using a commercially available ELISA (DRG Diagnostics, DRG International, Marburg, Germany), following the manufacturer's instructions. It was necessary to dilute 66-h samples 1 in 10 with the sample diluent provided to allow interpolation from the standard curve. hCG secretion was expressed as mIU/mg protein per h. Cell lysate protein content (mg) was determined using a commercial kit (Bio-Rad Laboratories Ltd.). Sixty-six hour data were analysed by Wilcoxon-signed Rank test following normalisation of *β*-hCG secretion by transfected cells to *β*-hCG secretion by the corresponding untransfected control cells for each experiment.

### Detection of apoptosis

Prior to methanol fixation at 66 h, transfected and non-transfected control cells were cultured overnight in ±100 ng TNF*α*. Apoptosis was determined by the presence of cleaved cytokeratin 18, detected by M30 CytoDEATH mouse monoclonal antibody (Roche Diagnostics Ltd, West Sussex, UK) using immunohistochemistry as follows: cells were washed with TBS then incubated for 10 min with 3% (v/v) H_2_O_2_ in distilled water to quench endogenous peroxidase. Following a second wash step, cells were incubated at room temperature for 30 min with blocking solution (10% normal goat serum, 2% human serum and 0.1% Tween in TBS). Primary antibody (1 : 100 dilution in blocking solution) or non-immune mouse IgG, included as a negative control, was then applied for 1 h at 37 °C. Unbound primary antibody was then removed by washing with TBS. Cells were incubated for 30 min at room temperature with biotinylated goat anti-mouse secondary antibody (Dako UK Ltd, Cambridge, UK, diluted 1 : 200 in blocking solution), washed again with TBS and then incubated for a further 30 min at room temperature with avidin peroxidase. Following another wash with TBS, cleaved cytokeratin 18 was detected by colour development with diaminobenzidine-hydrogen peroxide (DAB) and the cells were counterstained with Harris' haematoxylin. The percentage of apoptotic nuclei was then analysed using a Leitz Dialux 22 microscope and Image Pro Plus software. For each experiment, six field of view/areas of syncytia were analysed per treatment and each treatment was performed in duplicate. The mean from these 12 observations provided a measure of apoptotic nuclei for each treatment. Data were analysed using Kruskal–Wallis with Dunn's multiple comparison test.

## Abbreviations

BLAST: Basic Local Alignment Search Tool
FGR: foetal growth restriction
MAPK: mitogen-activated protein kinase
MVM: microvillous membrane
NT: non-targeting siRNA
PBS: phosphate buffered saline
PE: pre-eclampsia
PI: propidium iodide
PKC: protein kinase C
QPCR: quantitative polymerase chain reaction
RNS: reactive nitrogen species
siRNA: small interfering RNA
STAT3: signal transducer and activator of transcription 3
TBS: Tris buffered saline
TNF*α*: tumour necrosis factor alpha
